# Effects of Diets Containing Beef Compared with Poultry on Pancreatic β-Cell Function and Other Cardiometabolic Health Indicators in Males and Females with Prediabetes: A Randomized, Crossover Trial

**DOI:** 10.1016/j.cdnut.2025.107589

**Published:** 2025-10-30

**Authors:** Elizabeth Guzman, Indika Edirisinghe, Meredith L Wilcox, Carol F Kirkpatrick, Caryn G Adams, Britt M Burton-Freeman, Kevin C Maki

**Affiliations:** 1 Illinois Institute of Technology, Chicago, IL, United States; 2 Midwest Biomedical Research, Addison, IL, United States; 3 Kasiska Division of Health Sciences, Idaho State University, Pocatello, ID, United States; 4 Department of Applied Health Science, School of Public Health, Indiana University, Bloomington, IN, United States

**Keywords:** beef, biomarkers of inflammation, cardiometabolic risk factors, glucose homeostasis, lipoprotein lipids, pancreatic β-cell function, poultry, red meat, type 2 diabetes

## Abstract

**Background:**

The results of randomized controlled trials generally indicate that red meat intake, or beef specifically, has no impact on potential mechanisms that increase risk of type 2 diabetes, such as reduced insulin sensitivity. However, there have been limited studies conducted to assess the impact of red meat, including beef intake, on pancreatic β-cell function.

**Objectives:**

The purpose of this study was to examine the effects of beef intake compared with poultry intake on pancreatic β-cell function, other indicators of glucose homeostasis, glucoregulatory hormone responses, lipoprotein lipids, and biomarkers of inflammation in adults with prediabetes.

**Methods:**

This randomized crossover study included adults with overweight or obesity and prediabetes. The participants completed two 28-d treatment periods, separated by a 28-d washout period. Participants were provided entrées with beef or poultry (6–7 oz/d) to consume during the conditions and were otherwise instructed to maintain their habitual dietary pattern during the treatment and washout periods. The primary outcome was pancreatic β-cell function assessed as the ratio of the incremental areas under the curve (iAUC)_0–180 min_ for C-peptide to glucose. Secondary outcomes were fasting and postprandial levels of glucose, insulin, C-peptide, glucagon, glucagon-like peptide-1, glucose-dependent insulinotropic polypeptide, high-sensitivity C-reactive protein, fibrinogen, interleukin-6, and tumor necrosis factor-α. Effects on fasting lipoprotein lipid levels were an exploratory outcome.

**Results:**

Twenty-nine adults were randomized after screening for inclusion, of which 24 participants (17 males, 7 females) completed the trial. The results indicated no significant differences between beef and poultry for iAUC C-peptide_0–180 min_/iAUC glucose_0–180 min_ or any of the secondary and exploratory outcomes.

**Conclusions:**

Compared with poultry, consumption of beef for 28 d did not produce adverse effects on pancreatic β-cell function, other indicators of glucose homeostasis, lipoprotein lipids, or biomarkers of inflammation.

This trial was registered at clinicaltrials.gov as NCT05456477 (https://clinicaltrials.gov/study/NCT05456477).

## Introduction

Results from observational studies have shown associations of higher red meat intake with risk of incident type 2 diabetes mellitus (T2D) [[1], [2], [3], [4]], whereas most studies find no association or an inverse association with poultry intake [3]. Conversely, the results of randomized controlled trials (RCTs) generally report no impact of red meat intake on potential mechanisms and risk factors related to T2D [5,6]. Recent systematic reviews and meta-analyses of RCTs that examined the effects of red meat intake on risk factors related to T2D, including fasting glucose, fasting insulin, insulin sensitivity or resistance, and glycated hemoglobin (HbA1c), reported no significant impact of red meat intake on these parameters [6,7]. Thus, the mechanisms to explain the association of red meat intake and T2D suggested in observational studies, if causal, remain uncertain. It is possible that the association is related to residual confounding because individuals who consume high amounts of red meat often differ in other dietary and behavioral attributes that could influence risk of T2D [1,2,8], or there may be other mechanisms that have not been fully elucidated.

The development of T2D typically results from insulin resistance that occurs over an extended period, coupled with progressive deterioration of pancreatic β-cell function [[9], [10], [11]]. In the initial stages of insulin resistance, normoglycemia is maintained through compensatory hyperinsulinemia. However, this eventually contributes to pancreatic β-cell exhaustion and loss of function, resulting in insufficient insulin secretion to maintain normoglycemia [11]. Researchers have previously reported that the intake of red meat does not significantly alter insulin sensitivity [5,[12], [13], [14]], but fewer studies have been done to assess the impact of red meat intake on pancreatic β-cell function [6].

Glucose-dependent insulin secretion by the pancreatic β-cells is influenced by several variables, including the number of pancreatic β-cells, their sensitivity to glucose, and glucoregulatory hormones, such as glucagon-like peptide-1 (GLP-1) and glucose-dependent insulinotropic polypeptide (GIP) [[9], [10], [11]]. Chronic inflammation, diminished responses to glucoregulatory hormones, and increased SFA intake have been proposed as potential mechanistic factors connected to pancreatic β-cell dysfunction, and these mechanisms may be influenced by red meat intake [15,16]. The results of a previous study demonstrated that consuming a USDA Healthy Eating Pattern that included 150 g/d of lean beef did not significantly alter measures of pancreatic β-cell function, compared with the USDA Healthy Eating Pattern control diet that included <40 g/d of red meat [5]. However, in that study, β-cell function was measured during an intravenous glucose tolerance test, which cannot assess the effects of glucoregulatory hormones stimulated in response to food consumption. Furthermore, lean beef was substituted for refined starches in the dietary intervention of that study. Investigators in another study reported an improvement in pancreatic β-cell function when participants consumed a plant-based protein meal compared with a meal composed of processed red meat [17]. However, the test meals were also different in sugar, fiber, and unsaturated and SFA contents. Therefore, it is unclear what role, if any, the protein source may have had in the outcomes of that study.

The aim of the present study was to assess the effects of the dietary protein source (beef compared with poultry) on pancreatic β-cell function (primary outcome), glucose homeostasis, glucoregulatory hormone responses, and inflammation biomarker levels in individuals with prediabetes. The effects on lipoprotein lipids were examined as an exploratory outcome.

## Methods

### Study design

The study was a randomized, crossover trial conducted in accordance with Good Clinical Practice Guidelines, the Declaration of Helsinki, and the United States 21 Code of Federal Regulations [18]. An Institutional Review Board (Western Institutional Review Board-Copernicus Group) approved the protocol, and a signed informed consent form was obtained from all participants before study procedures were performed. Eligible participants were randomly assigned to a sequence using a computer-generated randomization scheme, and allocation was concealed in an envelope until the time of randomization. The randomization schedule used randomly permuted blocks with a block size of 2. Due to the nature of the conditions, participants and study staff were not able to be blinded to the interventions. The study consisted of one screening visit, a baseline visit during which participants were randomly assigned, and two 28-d treatment periods separated by a 28-d washout period. The first screening visit occurred on 7 October, 2022, the last participant was randomly assigned on 28 June, 2023, and the last participant visit occurred on 22 September, 2023.

### Participants

Eligible individuals included males and females, aged 18–74 y, with BMI ≥25.0 and <39.9 kg/m^2^^,^ and prediabetes (fasting plasma glucose of 100–125 mg/dL or HbA1c of 5.7%–6.4% at screening). Participants had to be in general good health and have adequate venous access. Additionally, they had to be willing to consume the study foods and avoid meat, poultry, seafood, and eggs (other than that provided in the study entrées) for the duration of the treatment periods. Exclusion criteria for the study included the presence of type 1 diabetes mellitus or T2D determined by self-report or a fasting blood glucose ≥126 mg/dL and/or HbA1c ≥6.5% at screening; a history of atherosclerotic cardiovascular disease, cancer (other than nonmelanoma skin cancer or carcinoma in situ of the cervix), or uncontrolled hypertension (systolic blood pressure ≥160 mm Hg and/or diastolic blood pressure ≥100 mm Hg) at screening; weight change ± 4.5 kg in the previous 3 mo; or any active infection requiring antibiotic therapy. Additional exclusion criteria included use of any of the following for 4 wk before screening: unstable use of antihypertensives, thyroid hormones, or lipid-altering medications; α- or β-adrenergic blockers, thiazide diuretics, diabetes medications, systemic corticosteroids, weight loss drugs, or dietary supplements impacting carbohydrate metabolism. Other exclusion criteria were restrictive dietary habits (e.g., vegetarian or vegan), a diagnosed eating disorder, an allergy or sensitivity to the study foods, or a current or recent history (previous 12 mo) of drug or alcohol abuse. Females of childbearing age who were pregnant, lactating, planning to become pregnant during the study period, or were unwilling to commit to the use of medically approved forms of contraception during the study were excluded. Any other condition the investigator believed would interfere with a potential participant’s ability to provide informed consent or comply was also exclusionary.

### Study procedures

Once eligibility was determined and informed consent was obtained at the screening visit (visit 1), relevant medical history from the last 5 y was obtained, including evaluation of prior and concomitant medications/dietary supplement use, and assessment of inclusion/exclusion criteria. Additional measurements were taken, including height, body weight, waist circumference, vein assessment, resting blood pressure, and heart rate. A urine pregnancy test was administered to all females aged <60 y. Participants were provided a 3-d diet record to complete before returning to the clinic for the baseline visit, and fasting blood samples were collected for chemistry profile and HbA1c measurements and archives. During both treatment periods, participants were instructed to maintain their habitual dietary pattern, with the exception of avoiding meat, eggs, seafood, and poultry, except for the study products. During the washout period, participants were instructed to maintain their habitual dietary pattern. Participants were encouraged to maintain their body weight for the duration of the study. Additionally, participants were asked to maintain their normal level of physical activity throughout the study and not to change their tobacco or nicotine use, except that they were instructed to avoid vigorous physical activity and abstain from nicotine, caffeine, recreational marijuana, and alcohol for 24 h before each clinic visit.

**FIGURE 1:**
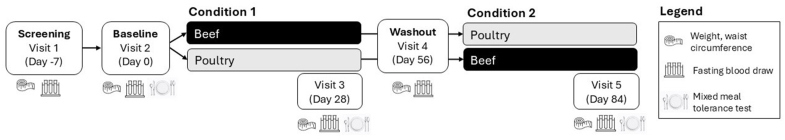
Summary of the visit procedures for the two 28-d diet conditions.

At the baseline visit (visit 2), participants arrived at the clinic after an overnight fast, and baseline data were obtained, including body weight, waist circumference, resting blood pressure, and heart rate (Figure 1). Premenopausal females were scheduled for test visits during the follicular phase of the menstrual cycle. The baseline 3-d diet record was collected and reviewed with the participant, and blood was collected for baseline levels of lipoprotein lipids (total cholesterol, calculated LDL cholesterol, HDL cholesterol, calculated non-HDL cholesterol, and triglycerides), and biomarkers of inflammation [high-sensitivity C-reactive protein (hs-CRP), IL-6, fibrinogen, and TNF-α]. A mixed meal tolerance test (MMTT) was then performed, during which individuals were given 15 min to consume a standardized breakfast meal. The meal included a plain white bagel (95 g), cream cheese (28 g), diced peaches (85 g), 2% milk (244 mL), and water or plain tea (237 mL), and contained 509 kcal (60% carbohydrate, 25% fat, 15% protein).

Blood samples were obtained before the consumption of the meal (−15 min) and at 30, 60, 120, and 180 min after consumption for measurement of glucose, insulin, C-peptide, glucagon, GLP-1, and GIP. Participants were then randomly assigned to a specific condition sequence and provided with study products containing either beef or poultry per the randomization assignment. After the 4-wk washout period, participants crossed over to the other treatment group (Figure 1).

At the conclusion of each condition and the washout (visits 3, 4, and 5), participants returned to the clinic after an overnight fast, and blood samples were collected to measure levels of lipoprotein lipids and biomarkers of inflammation. Measurements for body weight, waist circumference, blood pressure, and heart rate were also assessed. An MMTT was performed at the end of each condition (visits 3 and 5) using the same standardized breakfast and procedures as the baseline visit. Unused entrées and a 3-d diet record were collected at visits 3 and 5 to assess adherence to the intervention. Adverse events were assessed at each clinic visit (visits 2–5) using open-ended, nonleading questions. During visits with an MMTT, adverse events were assessed at the beginning and end of the visit.

### Study products

Entrées containing ∼3.0–3.5 oz cooked, unprocessed beef or poultry were prepared for the beef and poultry conditions, respectively. Five entrées were utilized within each condition to provide variety in meals. The entrées included fajitas, stew, burgers, burritos, and stir fry (Supplemental Table 1). Participants were provided approximately equal numbers of each type of entrée throughout both conditions. The quantity of beef or poultry to be included each day was based on the upper end of the range of actual consumption in the United States, and is similar to the amount provided in a previous study on insulin sensitivity and pancreatic β-cell function (5). The entrées were prepared by a catering service and were labeled and frozen for participants to take home. Participants received instructions on reheating the entrées and were instructed to consume two entrées each day, as part of their habitual dietary pattern, for the duration of the 28-d treatment period and to return any unused portions to the clinic.

### Plasma glucose, insulin, C-peptide levels, and indices of insulin sensitivity and pancreatic β-cell function

A laboratory at Illinois Institute of Technology (IIT) in the Department of Food Science and Nutrition analyzed plasma glucose (Cat #GL3815) and plasma insulin (Sekisui Diagnostics, United States Cat #KAI-071) using the Randox Daytona Automated Clinical Analyzer (Randox). Plasma C-peptide was analyzed by the University of Texas at Austin (UT Austin) using the Milliplex Map kit for human metabolic hormone panel (HMH3-34K, Millipore). Total AUC (tAUC) and incremental AUC (iAUC) were calculated for glucose, insulin, and C-peptide [19].

Insulin sensitivity and resistance were assessed with the Matsuda insulin sensitivity index (MISI) and HOMA-IR, respectively. MISI was calculated as 10,000/(*G*_0_ × *I*_0_ × *G*_mean_ × *I*_mean_)^0.5^, where *G*_0_ and *I*_0_ were fasting values for glucose (mg/dL) and insulin (μIU/mL), respectively, and *G*_mean_ and *I*_mean_ were the respective mean postmeal values of glucose and insulin computed across the whole test as the tAUC divided by 120 min, where the tAUC was computed using the *t* = 0, 60, 120 min time points [20,21]. HOMA-IR was calculated using the equation: [fasting insulin (μIU/mL) × fasting plasma glucose (mg/dL)]/405 [22,23].

Pancreatic β-cell function was assessed by calculating the ratio of the iAUC for C-peptide to iAUC for glucose (primary outcome) and the tAUC for C-peptide to tAUC for glucose from pre-MMTT to 180 min after the start of food consumption for the MMTT [24]. Homeostasis model assessment of pancreatic β-cell function (HOMA-β) and the disposition index were also calculated as additional measures of pancreatic β-cell function. HOMA-β using fasting insulin was calculated using the equation: fasting insulin (μIU/mL) × 360/[fasting plasma glucose (mg/dL) − 63] [22,25], and HOMA-β using fasting C-peptide was calculated using the equation: 0.27 × {[fasting C-peptide (pmol/L)]/[fasting plasma glucose (mmol/L) − 3.5]} [26]. The disposition index was calculated using insulin as MISI × (tAUC insulin/tAUC glucose) and using C-peptide as MISI × (tAUC C-peptide/tAUC glucose) [27,28].

### Glucoregulatory hormones

Glucagon, GLP-1, and GIP were analyzed at UT Austin using Milliplex Map kit for human metabolic hormone panel (HMH3-34K, Millipore), and tAUC and iAUC were calculated for these hormones.

### Lipoprotein lipids and biomarkers of inflammation

IIT analyzed total cholesterol (Randox Cat # CH 3810), HDL cholesterol (Randox Cat # CH 3811), and total triglyceride (Randox Cat # TR 3823) levels using the Randox Daytona Automated Clinical Analyzer (Randox) with appropriate standards and quality controls. LDL cholesterol was calculated using the Friedewald equation [29], and non-HDL cholesterol was calculated as total cholesterol – HDL cholesterol. Plasma hs-CRP levels were assessed by IIT using an immunoturbidimetric method deployed by the Randox Automated Clinical Analyzer (Cat #CP 3885). IL-6 (Cat #SS600C) and TNF-α (Cat #SSTA00E) were assessed by IIT using sandwich ELISA methods using R&D Systems assay kits. Fibrinogen was assessed by Edward-Elmhurst Reference Laboratory with quantitative clot-based detection (STA-Fibrinogen 5 kit) using the Stago Analyzer.

### Diet records and adherence

Participants completed written 3-d diet records, including two weekdays and one weekend day, at baseline and during each treatment period. Diet records were analyzed using Food Processor Nutrition Analysis & Fitness Software (version 11.11.32, ESHA Research). Adherence to consumption of study entrées was assessed by counting the number of uneaten study entrées returned by participants at visits 3 and 5 (end of each condition) to calculate the percentage of expected servings consumed. Adherence was also assessed by reviewing daily food logs with participants and querying them regarding any unreturned entrées that may not have been consumed. Adequate adherence for each condition was defined as >80% to <120% of expected servings consumed.

### Statistical analysis

An evaluable sample size of 24 participants was expected to provide 80% power to detect a difference between conditions of 0.6 SDs for the primary outcome. The primary analysis included data from all participants who were randomly assigned and provided ≥1 postrandomization outcome data point during each treatment period (evaluable analysis sample). The per-protocol analysis sample is a subset of the evaluable analysis sample that excludes participants with protocol deviations (e.g., violation of inclusion/exclusion criteria, nonadherence with study product, missing clinic visits, etc.). The safety analysis sample includes all participants who were randomly assigned into the study and consumed ≥1 dose of study product.

The numbers of participants and percentages are reported for categorical variables. Means (SEM) are reported for continuous variables for baseline characteristics. Differences between conditions were assessed using repeated measures analysis of covariance. Each model used the baseline value as a covariate and participant nested within sequence as a random effect. Sensitivity analyses were performed to test for possible treatment-by-period/sequence interactions, and none were observed in the evaluable sample analyses. Assumptions of normality of residuals were investigated for each outcome. Because several variables showed non-normal distributions as assessed with a quantile–quantile plot, natural logarithm transformations were used, and results for cardiometabolic variables are presented as geometric means for baseline and least squares geometric means (LSGM) for the end of each diet condition. Tests of significance were performed at α = 0.05, 2-sided, unless otherwise specified. All analyses were conducted using SAS, version 9.4 (SAS Institute Inc.) or R software version 4.4.2 (R Foundation). In selected instances, interpolation was used to estimate missing postprandial values. A sensitivity analysis with multiple imputations was completed to account for missing data from participants who dropped out of the study, and the results were not materially different; therefore, only the analysis results for the evaluable sample are presented.

## Results

**FIGURE 2:**
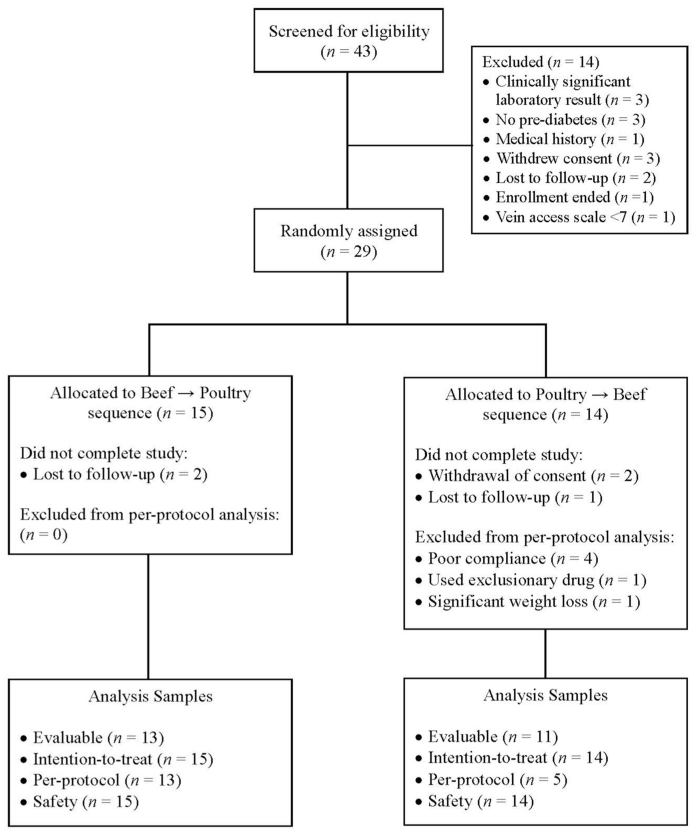
Flow diagram of participants assessed for eligibility, excluded, randomly assigned, and analyzed for the study.

A total of 43 participants were screened, of whom 29 were randomly assigned. The reasons for screen failure, discontinuation of the study, and protocol deviations are summarized in Figure 2. Evaluable data were available for 24 participants (17 males and 7 females) who completed the trial. Baseline characteristics for the evaluable analysis sample are presented in Table 1.

**TABLE 1:** Baseline characteristics of participants in the evaluable analysis sample according to condition sequence and for the overall sample

Characteristics^1^	Beef → Poultry (*n* = 13)	Poultry → Beef (*n* =11)	Overall (*n* = 24)
Age (y)	49.0 (4.15)	43.3 (3.72)	46.4 (2.82)
Sex			
Female, *n* (%)	5 (38.5)	2 (18.2)	7 (29.2)
Male, *n* (%)	8 (61.5)	9 (81.8)	17 (70.8)
Race			
Asian, *n* (%)	5 (38.5)	2 (18.2)	7 (29.2)
Black/African American, *n* (%)	3 (23.1)	2 (18.2)	5 (20.8)
White/Caucasian, *n* (%)	4 (30.8)	7 (63.6)	11 (45.8)
Not disclosed, *n* (%)	1 (7.7)	0 (0)	1 (4.2)
Ethnicity			
Hispanic/Latino, *n* (%)	0 (0)	4 (36.4)	4 (16.7)
Not Hispanic/Latino, *n* (%)	13 (100)	7 (63.6)	20 (83.3)
Smoking status			
Never, *n* (%)	9 (69.2)	7 (63.6)	16 (66.7)
Current, *n* (%)	1 (7.7)	0 (0)	1 (4.2)
Former, *n* (%)	3 (23.1)	4 (36.4)	7 (29.2)
Alcohol use			
None, *n* (%)	4 (30.8)	4 (36.4)	8 (33.3)
Occasionally, *n* (%)	7 (53.8)	3 (27.3)	10 (41.7)
Weekly, *n* (%)	2 (15.4)	4 (36.4)	6 (25.0)
Weight (kg)	83.0 (1.78)	92.4 (6.20)	87.3 (3.08)
BMI (kg/m^2^)	28.9 (0.61)	31.1 (1.57)	29.9 (0.81)
Waist circumference (cm)	96.5 (2.19)	102 (5.40)	99.1 (2.74)
Systolic blood pressure (mm Hg)	123 (4.08)	122 (4.51)	123 (2.96)
Diastolic blood pressure (mm Hg)	79.0 (2.61)	74.9 (2.75)	77.1 (1.90)
HbA1c (%)	5.52 (0.09)	5.19 (0.10)	5.37 (0.07)

Abbreviation: HbA1c, glycated hemoglobin.

1 For continuous variables, values are shown as mean (SEM).

Medians and quartile thresholds (Q1, Q3) for energy and selected nutrient intakes for the evaluable analysis sample at baseline are provided in Table 2. The median (Q1, Q3) baseline energy intake was 1648 (1466, 2218) kcal/d. It has been shown in previous studies that the energy intake estimated from diet records is underestimated by ∼20% [30]. This was the case for the participants in the current study, whose estimated energy intake at baseline was ∼79% of the median calculated energy intake during the beef and poultry conditions.

**TABLE 2:** Energy, macronutrient, and selected nutrient intakes at baseline^1^

Variables	Baseline median (Q1, Q3)
Energy (kcal/d)	1648 (1466, 2218)
Carbohydrates (% energy)	48.0 (40.5, 51.5)
Sugars (% energy)	16.8 (9.26, 20.8)
Dietary fiber (g/d)	14.3 (11.2, 22.9)
Proteins (% energy)	16.1 (13.6, 20.8)
Dietary fats (% energy)	37.1 (32.8, 42.4)
UFAs (% energy)	23.7 (20.8, 29.1)
SFAs (% energy)	12.7 (10.4, 13.8)
Cholesterol (mg/d)	247 (137, 386)
Sodium (mg/d)	2794 (2066, 3481)

Abbreviations: Q1, first quartile (25th percentile); Q3, third quartile (75th percentile); UFAs, unsaturated fatty acids.

1 Sample size: *n* = 24.

Median (Q1, Q3) values for energy and selected nutrient intakes for the evaluable analysis sample at the end of each condition are provided in Table 3. There were significant differences between the beef and poultry conditions for some of the macronutrients and nutrients. During the poultry condition, there were higher percentages of total energy intake from carbohydrates and proteins, whereas during the beef condition, there were higher percentages of total energy from sugars, total dietary fats, and SFAs.

**TABLE 3:** Energy, macronutrient, and selected nutrient intakes at the end of condition for each condition sequence from 3-d diet record analysis^1^

Variables	Beef Median (Q1, Q3)^2^	Poultry Median (Q1, Q3)^2^	*P* value^3^
Energy (kcal/d)	2079 (1642, 2446)	2080 (1562, 2374)	0.881
Carbohydrates (% energy)	45.9 (40.5, 50.0)	47.1 (42.3, 50.6)	0.016
Sugars (% energy)	12.3 (9.68, 17.6)	10.9 (9.16, 18.2)	0.023
Dietary fiber (g/d)	17.8 (15.2, 25.4)	20.9 (15.8, 26.3)	0.711
Proteins (% energy)	20.1 (16.2, 21.2)	21.1 (18.8, 23.9)	0.015
Dietary fats (% energy)	35.8 (32.6, 38.9)	33.1 (28.8, 35.5)	<0.001
UFAs (% energy)	24.4 (20.8, 27.2)	24.4 (20.4, 26.5)	0.188
SFAs (% energy)	11.6 (9.08, 12.6)	8.34 (7.22, 10.5)	<0.001
Cholesterol (mg/d)	224 (188, 248)	225 (177, 269)	0.824
Sodium (mg/d)	3572 (2988, 4413)	3597 (2894, 4514)	0.928

Abbreviations: Q1, first quartile (25th percentile); Q3, third quartile (75th percentile); UFAs, unsaturated fatty acids.

1 Sample size: *n* = 24 for each condition. Data are the averages from the 3-d diet records. If <3 d were completed, the average of the available days was used.

2 Medians (Q1, Q3) are presented for all variables because some had skewed distributions.

3 *P* values for differences between conditions in end-of-treatment values were generated from a linear mixed model that included end-of-treatment values as the dependent variable, condition as a fixed effect, and participant as a random effect.

The median (Q1, Q3) percent adherence with consumption of the study products was 99.1% (94.8%, 100%) during the beef condition and 100% (96.2%, 100%) during the poultry condition (*P* = 0.20 between conditions). Two participants reported a treatment-emergent adverse event, both of which were judged to be unrelated to the study foods.

### Plasma glucose, insulin, C-peptide, and glucoregulatory hormones levels

**FIGURE 3:**
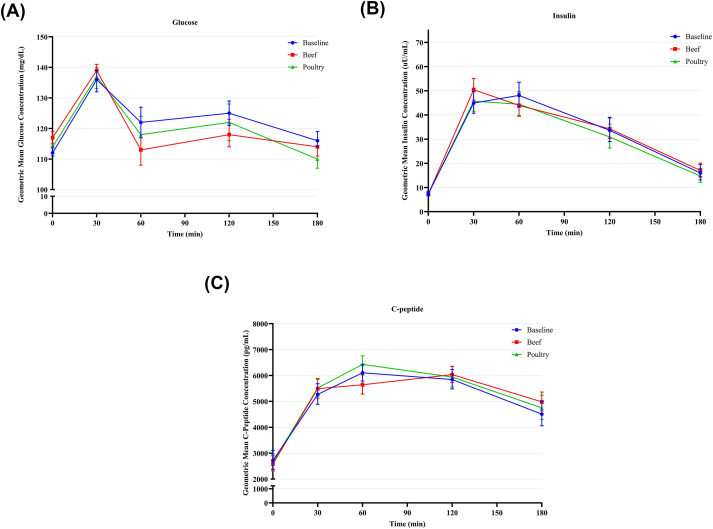
Geometric mean (−SE, +SE) concentrations by condition and time point for glucose (A), insulin (B), and C-peptide (C). The line graphs illustrate the results for glucose (A), insulin (B), and C-peptide (C) at timepoints 0, 60, 120, and 180 min during the mixed meal tolerance test at baseline and the end of each condition.

The geometric mean (−SE, +SE) concentrations by condition and time point for glucose, insulin, and C-peptide are shown in Figure 3(A–C). The results for fasting glucose, insulin, C-peptide, and glucoregulatory hormone levels, as well as tAUC_0–180 min_ and iAUC_0–180 min_ results for these variables, at baseline and the end of each condition for the evaluable analysis sample, are summarized in Table 4. The tAUC_0–120 min_ and iAUC_0–120 min_ results for glucose, insulin, and the glucoregulatory hormones at baseline and the end of each condition for the evaluable analysis sample are summarized in Supplemental Table 2. For the primary outcome, there was no significant difference between the beef and poultry conditions for iAUC_0–180 min_ C-peptide:iAUC_0–180 min_ glucose. There were no significant differences between the beef and poultry conditions for any other glucose homeostasis parameters or glucoregulatory hormone parameters. The results for the per-protocol analysis sample were similar to those of the evaluable analysis sample, except for a significant difference between the beef and poultry conditions for tAUC_0-120 min_ for C-peptide {LSGM for beef end-of-condition: 639 [min × (pg/mL)] × 10^−3^] (95% confidence interval: 587, 696); LSGM for poultry end-of-condition: 693 [min × (pg/mL)] × 10^−3^] (95% confidence interval: 636, 754); *P* = 0.026}.

**TABLE 4:** Glucose, insulin, C-peptide, and glucoregulatory hormone parameters at baseline and end of each condition in the evaluable analysis sample^1^

Variables	Baseline^2^	Beef^3^	Poultry^3^	*P* value^4^
Fasting glucose (mg/dL)	112 (104, 121)	117 (114, 120)	114 (111, 117)	0.172
tAUC_0–180 min_ glucose [min × (mg/dL)]	22,328 (19,768, 25,218)	21,623 (20,725, 22,560)	21,908 (20,998, 22,857)	0.594
iAUC_0–180 min_ glucose [min × (mg/dL)]	1563 (398, 6141)	747 (362, 1540)	1023 (496, 2111)	0.534
Fasting insulin (μIU/mL)	7.41 (4.19, 13.1)	7.31 (6.50, 8.22)	7.37 (6.56, 8.29)	0.890
tAUC_0–180 min_ insulin [min × (μIU/mL)]	6477 (3860, 10,867)	6439 (5732, 7234)	6185 (5506, 6949)	0.480
iAUC_0–180 min_ insulin [min × (μIU/mL)]	5017 (2857, 8810)	5005 (4356, 5751)	4773 (4154, 5484)	0.480
Fasting C-peptide (pg/mL)	2719 (1418, 5212)	2588 (2152, 3113)	2652 (2205, 3190)	0.664
tAUC_0–180 min_ C-peptide [min × (pg/mL)] × 10^−3^]	977 (726, 1315)	988 (931, 1049)	1006 (948, 1068)	0.554
iAUC_0–180 min_ C-peptide [min × (pg/mL)] × 10^−3^]	357 (97.6, 1304)	470 (413, 534)	477 (419, 542)	0.854
tAUC C-peptide_0–180 min_/tAUC glucose_0–180 min_, {[min × (pg/mL)]/[min × (mg/dL)]}	43.8 (33.3, 57.4)	45.7 (43.0, 48.6)	45.9 (43.1, 48.9)	0.880
iAUC C-peptide_0–180 min_/iAUC glucose_0–180 min_, {[min × (pg/mL)]/[min × (mg/dL)]}	216 (38.7, 1202)	478 (301, 759)	366 (230, 581)	0.249
Fasting glucagon (pg/mL)	112 (38.0, 327)	98.1 (66.2, 145)	109 (73.9, 162)	0.597
tAUC_(0–180 min)_ glucagon [min × (pg/mL)] × 10^−3^]	34.6 (15.7, 76.2)	31.2 (23.1, 42.1)	36.8 (27.3, 49.7)	0.184
iAUC_0–180 min_ glucagon {[min × (pg/mL)] × 10^−3^}	10.5 (3.44, 32.2)	10.6 (7.45, 15.0)	13.1 (9.22, 18.6)	0.313
Fasting GLP-1 (pg/mL)	322 (141, 735)	291 (232, 366)	357 (284, 449)	0.072
tAUC_0–180 min_ GLP-1 {[min × (pg/mL)] × 10^−3^}	78.4 (38.3, 160)	79.3 (66.0, 95.3)	90.4 (75.3, 109)	0.157
iAUC_0–180 min_ GLP-1 {[min × (pg/mL)] × 10^−3^}	15.5 (3.72, 64.7)	19.1 (13.4, 27.2)	20.6 (14.5, 29.4)	0.623
Fasting GIP (pg/mL)	872 (459, 1656)	777 (617, 980)	811 (643, 1022)	0.726
tAUC_0–180 min_ GIP {[min × (pg/mL)] × 10^−3^}	676 (492, 928)	665 (621, 713)	686 (640, 735)	0.461
iAUC_0–180 min_ GIP {[min × (pg/mL)] × 10^−3^}	389 (102, 1490)	506 (433, 592)	485 (414, 567)	0.618

Abbreviations: CI, confidence interval; GIP, glucose-dependent insulinotropic polypeptide; GLP-1, glucagon-like peptide-1; GM, geometric mean; iAUC, incremental AUC; LSGM, least squares geometric mean; tAUC, total AUC.

1 Sample size: *n* = 24 for each condition for all variables, except iAUC C-peptide_0–180 min_/iAUC glucose_0–180 min_ (*n* = 22), which was the primary outcome. Two participants had an end-of-condition glucose iAUC value of zero and were excluded from the iAUC C-peptide_0–180 min_/iAUC glucose_0–180 min_ analysis.

2 Results reported are GM (−1 SD, +1 SD).

3 Results reported are LSGM (95% CI) for end-of-condition.

4 *P* values are for beef vs. poultry using repeated measures analysis of covariance, with baseline as a covariate. No significant condition-by-period interactions were present.

### Insulin sensitivity, glucose homeostasis, and pancreatic β-cell function

The results for insulin sensitivity (MISI, HOMA-IR) and pancreatic β-cell function (HOMA-β) at baseline and the end of each treatment sequence are summarized in Table 5 [20,21,27,28]. There were no significant differences between the beef and poultry conditions for any of the insulin sensitivity or pancreatic β-cell function parameters in the evaluable analysis sample. The results for the per-protocol analysis sample were similar.

**TABLE 5:** Insulin sensitivity, indices of glucose homeostasis, and pancreatic β-cell function parameters at baseline and end of each condition in the evaluable analysis sample^1^

Variables	Baseline^2^	Beef^3^	Poultry^3^	*P* value^4^
MISI^5^	5.31 (3.04, 9.28)	5.55 (4.90, 6.28)	5.52 (4.87, 6.25)	0.945
Disposition index, insulin^6^	1.71 (1.22, 2.38)	1.82 (1.66, 2.00)	1.71 (1.56, 1.88)	0.172
Disposition index, C-peptide^7^	233 (145, 373)	247 (216, 282)	252 (220, 288)	0.806
HOMA-IR	2.06 (1.14, 3.72)	2.11 (1.86, 2.40)	2.08 (1.83, 2.36)	0.803
HOMA-β	3.03 (1.74, 5.26)	2.72 (2.42, 3.06)	2.93 (2.61, 3.30)	0.302

Abbreviations: CI, confidence interval; GM, geometric mean; HOMA-β, homeostasis model assessment of pancreatic β-cell function; LSGM, least squares geometric mean; MISI, Matsuda insulin sensitivity index.

1 Sample size: *n* = 24 for each condition.

2 Results reported are GM (−1 SD, +1 SD).

3 Results reported are LSGM (95% CI) for end-of-condition.

4 *P* values are for beef vs. poultry using repeated measures analysis of covariance, with baseline as a covariate. No significant condition-by-period interactions were present.

5 Calculated as 10,000/(*G*_0_ × *I*_0_ × *G*_mean_ × *I*_mean_)^0.5^, where *G*_0_ and *I*_0_ were fasting values for glucose (mg/dL) and insulin (μIU/mL), respectively, and *G*_mean_ and *I*_mean_ were the respective mean postmeal values of glucose and insulin computed across the whole test as the total AUC divided by 120 min, where the total AUC was computed using the *t* = 0, 60, 120 min time points [20,21].

6 Calculated using insulin (MISI × tAUC insulin/tAUC glucose) [27,28].

7 Calculated using C-peptide (MISI × tAUC C-peptide/tAUC glucose) [27,28].

### Lipoprotein lipids and biomarkers of inflammation

The results for lipoprotein lipids and biomarkers of inflammation at baseline and the end of each condition are summarized in Table 6. There were no significant differences between the beef and poultry conditions for any of these parameters in the evaluable analysis sample, and the per-protocol analysis sample results were similar.

**TABLE 6:** Lipoprotein lipids and biomarkers of inflammation at baseline and end of each condition in the evaluable analysis sample^1^

Variables	Beef baseline^2^	Beef^3^	Poultry baseline^2^	Poultry^3^	*P* value^4^
Total cholesterol (mg/dL)	176 (143, 216)	175 (167, 183)	176 (145, 213)	173 (165, 181)	0.657
LDL cholesterol (mg/dL)	113 (87.6, 146)	112 (104, 120)	113 (88.3, 145)	112 (105, 121)	0.887
HDL cholesterol (mg/dL)	44.2 (35.5, 54.9)	45.1 (43.4, 47.0)	44.5 (35.6, 55.6)	43.1 (41.4, 44.9)	0.117
Non-HDL cholesterol (mg/dL)	130 (101, 168)	128 (120, 136)	129 (101, 166)	128 (120, 137)	0.964
Triglycerides (mg/dL)	78.6 (49.6, 125)	76.1 (66.4, 87.2)	77.1 (50.2, 118)	74.5 (64.9, 85.4)	0.747
hs-CRP (mg/L)	2.31 (0.47, 11.3)	1.79 (0.97, 3.29)	2.26 (0.60, 8.53)	1.59 (0.87, 2.92)	0.393
IL-6 (pg/mL)	1.71 (0.69, 4.27)	1.47 (1.09, 1.99)	1.62 (0.76, 3.44)	1.44 (1.06, 1.94)	0.852
Fibrinogen (mg/dL)	291 (202, 421)	267 (238, 299)	267 (196, 365)	255 (227, 285)	0.558
TNF-α (pg/mL)	0.76 (0.56, 1.03)	0.70 (0.64, 0.76)	0.73 (0.56, 0.97)	0.70 (0.64, 0.76)	0.933

Abbreviations: CI, confidence interval; GM, geometric mean; hs-CRP, high-sensitivity C-reactive protein; LSGM, least squares geometric mean.

1 Sample size: *n* = 24 for each condition.

2 Results reported are GM (−1 SD, +1 SD).

3 Results reported are LSGM (95% CI) for end-of-condition.

4 *P* values are for beef vs. poultry using repeated measures analysis of covariance, with baseline as a covariate. No significant condition-by-period interactions were present.

## Discussion

The results of this study indicate that, compared with poultry consumption, there were no differences in measures of pancreatic β-cell function after 28-d of consuming 6–7 oz/d of unprocessed beef, when incorporated into participants’ habitual dietary patterns. Additionally, beef consumption did not impact other measures of cardiometabolic health, including indicators of glucose homeostasis, glucoregulatory hormones, lipoprotein lipids, or biomarkers of inflammation, compared with poultry intake.

The results of this study are consistent with the results of systematic reviews and meta-analyses conducted to examine the effects of red meat intake on glycemic control and inflammation biomarkers [6,7]. Sanders et al. [6] conducted a meta-analysis of 21 RCTs that compared red meat (beef, pork, lamb, etc.) intake with reduced or no red meat intake in adults. The participants in the RCTs included in the meta-analysis were healthy or had metabolic syndrome or T2D. The results of the meta-analysis indicated that there was no significant impact of red meat intake on fasting glucose, fasting insulin, GLP-1 levels, insulin sensitivity, insulin resistance, or pancreatic β-cell function, compared with reduced or no red meat consumption. It is important to note that there was a limited number of studies that examined the effects of red meat intake, compared with reduced meat or no meat intake, on pancreatic β-cell function (3 RCTs) and GLP-1 levels (4 RCTs). Therefore, the current study adds to the limited available evidence suggesting that unprocessed red meat intake does not adversely impact pancreatic β-cell function.

O’Connor et al. [7] included 20 RCTs in a meta-analysis to examine the effects of total red meat (mainly unprocessed beef and pork) intake on glycemic control and inflammation biomarkers in adults without cardiometabolic disease but at risk for cardiovascular disease or T2D. The results of their meta-analysis demonstrated that there was no significant difference between total red meat intake ≥0.5 servings/d compared with <0.5 servings/d for fasting glucose, insulin, HOMA-IR, HbA1c, CRP, IL-6, or TNF-α parameters. In a subgroup analysis, the authors examined the effect of replacing red meat with other animal-based proteins, such as poultry, and found no difference between total red meat and other replacement proteins. This finding is consistent with the results of the current study, which showed no significant differences in glucose homeostasis parameters and biomarkers of inflammation between beef and poultry consumption. The duration of the RCTs in the meta-analyses discussed above ranged from 3 to 16 wk; therefore, studies of longer duration would be beneficial to examine the effects of red meat intake on glycemic control, insulin sensitivity, and biomarkers of inflammation [6,7].

The results for fasting glucose and insulin levels in the current study were similar to the results found in a randomized, crossover, controlled feeding trial in which participants (*n* = 41) received a Mediterranean-style eating pattern with either ∼500 g/wk red meat (unprocessed lean beef and pork) or ∼200 g/wk red meat, with poultry being the primary protein food to compensate for the lower red meat content, for 5 wk each with a 4-wk washout period [13]. In that study, there were no significant differences between the two dietary interventions for fasting glucose and insulin levels. Additionally, researchers conducted an RCT to examine the effects of the Dietary Approaches to Stop Hypertension dietary pattern with either 28 g lean beef/d, 113 g lean beef/d, or 153 g lean beef/d compared with a healthy American diet on various cardiometabolic risk factors, including fasting glucose, insulin, and CRP levels [31]. At the end of 5 wk on each diet condition, there were no significant differences between any of the dietary interventions for glucose, insulin, and CRP levels.

The results for lipoprotein lipid levels in the current study are similar to those from a previous RCT that examined the effects of lean red meat on lipoprotein lipids in males and females with hypercholesterolemia, compared with lean white meats [32]. After participants consumed a National Cholesterol Education Program Step 1 diet with either ∼136 g lean red meat (beef, pork, or lamb) (*n* = 89) or white meat (poultry or fish) (*n* = 102) 5–7 d/wk for 36 wk, the effects on lipoprotein lipid levels were nearly identical, with a similar reduction in total and LDL cholesterol and an increase in HDL cholesterol levels in both study groups. Following a 4-wk washout period, the participants crossed over to the other diet intervention [i.e., the lean red meat group → lean white meat (*n* = 72) and lean white meat group → lean red meat (*n* = 73)] for another 36 wk. At the end of the second phase of the study, the effects on the lipoprotein lipids were the same as those observed at the end of the first phase, with no significant differences in any of the lipoprotein lipid levels between the diet interventions and similar reductions in total and LDL cholesterol and an increase in HDL cholesterol in both study groups [33]. Additionally, a meta-analysis of 27 RCTs examining the effects of diets containing fresh, unprocessed, or minimally processed beef, compared with a control diet without beef or with a lower amount of beef, yielded results similar to those of the current study [34]. Compared with the control diets, beef consumption did not impact total cholesterol, HDL cholesterol, non-HDL cholesterol, or triglycerides. There was a small but significant increase in LDL cholesterol; however, this became nonsignificant when one RCT was removed in a sensitivity analysis [34].

The results of the current study and previous RCTs suggest that beef can be included in an overall healthy dietary pattern without negatively impacting cardiometabolic risk factors, including risk factors for T2D. This is contrary to the results from observational studies, which have shown an association between red meat consumption and incident T2D [1,2,4]. However, several observational studies have shown that there are lifestyle differences between individuals who consume more compared with less red meat that may confound those results. Individuals who consume higher amounts of red meat are more likely to consume fewer fruits, vegetables, whole grains, and fiber; consume higher amounts of alcohol and foods rich in SFAs and added sugars; and to be less active, current smokers, and have a higher BMI [1,2,6,7].

The strengths of this study included a high self-reported adherence with the consumption of the study products (99%–100%) and measurement of multiple glucoregulatory hormone responses. Limitations include the inability to blind research personnel and participants to the interventions, the relatively short duration of the diet conditions, the lack of objective confirmation of self-reported study product adherence, and that the results are only generalizable to unprocessed beef and poultry. Additionally, the participants were free living during the study. Therefore, although beef and poultry entrées were provided to the participants, the other foods they consumed during the study were not fully controlled. Studies of longer duration would be beneficial to examine the effects of red meat intake on glycemic control, insulin sensitivity, glucoregulatory hormones, lipoprotein lipids, and biomarkers of inflammation to determine whether any differences emerge over longer periods. Additionally, glucose homeostasis was only assessed in the fasting state and in response to a standard mixed meal after an overnight fast at the end of each diet condition. Additional studies with continuous glucose monitoring would help to further clarify the effects of different types of meat consumption throughout the day and overnight. Finally, participants in this study had prediabetes and were predominantly male; thus, the generalizability to females and people without prediabetes or with diabetes is uncertain.

In conclusion, the results of this study suggest that consuming beef does not impact pancreatic β-cell response, glucose homeostasis, glucoregulatory hormones, lipoprotein lipids, or biomarkers of inflammation, compared with consuming poultry, in participants with prediabetes. These results contribute to the body of evidence suggesting that short-term, unprocessed beef intake does not impact risk factors for T2D and that unprocessed beef can be consumed, ideally as part of an overall healthy dietary pattern, without adversely affecting the cardiometabolic risk factor profile. Future RCTs are needed to investigate the effects of beef intake over longer periods on the cardiometabolic risk factor profile in a broader metabolically impaired population.

## Author contributions

The authors’ responsibilities were as follows – KCM, EG, IE, BMBF: designed and conducted research; IE: provided essential reagents or materials; MLW: analyzed data; MLW, CFK, CGA, KCM: wrote the first draft of the article; KCM: had primary responsibility for the final content; and all authors: reviewed and edited the article, and read and approved the final manuscript.

## Data availability

Data described in the manuscript, code book, and analytic code will be made available upon request, pending application and approval of the request.

## Funding

This research was funded by the National Cattlemen’s Beef Association, a contractor to the Beef Checkoff. The sponsor was allowed to comment on the study design as part of the application process. The sponsor had no role or involvement in the collection, analysis, or interpretation of the data; in the writing of the manuscript; or regarding the submission of the manuscript for publication regardless of the results of the study.

## Conflict of interest

MLW is an employee of Midwest Biomedical Research, which has received research funding and consulting fees from food and pharmaceutical companies. CFK is an employee of Midwest Biomedical Research, which has received research funding and consulting fees from food and pharmaceutical companies. CGA is an employee of Midwest Biomedical Research, which has received research funding and consulting fees from food and pharmaceutical companies. BMBF has received research grant support from the California Strawberry Commission, Gallo Inc., Hass Avocado Board, National Institutes of Health/Nutrition for Precision Health Common Fund, National Mango Board, USDA/National Institute of Food and Agriculture, and the Watermelon Promotion Board; received honoraria for lectures from the National Mango Board, Today’s Dietitian, and the University of Missouri; and served on advisory boards for the McCormick Science Institute, the Nutrient Institute, and NutriSciences Innovation, LLC. KCM has received research grant support from Cargill, General Mills, Global Organization for EPA and DHA, Greenyn Biotechnology, Hass Avocado Board, Helaina, Inc., Indiana University Foundation, Matinas BioPharma, MDLifespan, Medifast, Inc., National Cattlemen’s Beef Association/Beef Checkoff, National Dairy Council, Naturmega, NewAmsterdam Pharma, Novo Nordisk, PepsiCo, Pharmavite, and Ro; and received consulting fees from and/or served on advisory boards of 89bio, Acasti Pharma, Beren Therapeutics, Bragg Live Food Products, Campbell’s Company, Eli Lilly and Company, Esperion Therapeutics, Inc., Helaina, Inc., Lonza Group, Matinas BioPharma, MDLifespan, National Cattlemen’s Beef Association, National Dairy Council, NewAmsterdam Pharma, NorthSea Therapeutics, Novo Nordisk, and Seed Inc. If there are other authors, they declare that they have no known competing financial interests or personal relationships that could have appeared to influence the work reported in this paper.
