# Supplementary material for: Effects of Diets Containing Beef Compared with Poultry on Pancreatic β-Cell Function and Other Cardiometabolic Health Indicators in Males and Females with Prediabetes: A Randomized, Crossover Trial
Source: Curr Dev Nutr. 2025 Oct 30;9(12):107589. doi: 10.1016/j.cdnut.2025.107589 (PMC12686909; doi:10.1016/j.cdnut.2025.107589)
Supplement: multimedia component 1 [file mmc1.pdf]

Supplementary Material

Supplement to:

Effects of diets containing beef compared with poultry on pancreatic β-cell function and other cardiometabolic health indicators in males and females with prediabetes: a randomized, crossover trial

Elizabeth Guzman, MS[1], Indika Edirisinghe, PhD[1], Meredith L. Wilcox, MPH[2], Carol F. Kirkpatrick, PhD, MPH, RDN[2,3], Caryn G. Adams, MPH[2], Britt M. Burton-Freeman, PhD[1], Kevin C. Maki, PhD[2,4]*

[1]Department of Food Science and Nutrition, Illinois Institute of Technology, Chicago, IL 60616, USA
[2]Midwest Biomedical Research, Addison, IL 60101, USA
[3]Kasiska Division of Health Sciences, Idaho State University, Pocatello, ID 83209, USA
[4]Department of Applied Health Science, School of Public Health, Indiana University, Bloomington, IN 47401, USA

***Corresponding Author**
Kevin C. Maki, PhD
Midwest Biomedical Research
211 East Lake St., Ste. 3, Addison, IL 60101
Email: kmaki@mbclinicalresearch.com

Supplemental Table 1. Entrées provided to study participants during beef and poultry conditions.

	Beef Study Foods					Poultry Study Foods				
**Variables**	**Fajitas**	**Stew**	**Burger**	**Burrito**	**Stir Fry[1]**	**Fajitas**	**Stew**	**Burger**	**Burrito**	**Stir Fry[1]**
Calories	424	335	523	701	274	415	330	465	669	249
Total carbohydrates, g	31.6	24.2	43.3	81.3	7.95	31.6	24.2	43.3	81.3	7.95
Fiber, g	2.30	3.74	3.29	7.16	2.84	2.30	3.74	3.29	7.16	2.84
Sugars, g	3.89	6.72	10.4	3.25	3.85	3.89	6.72	10.4	3.25	3.85
Total protein, g	30.9	36.1	22.5	38.0	29.1	30.3	41.9	27.8	39.6	29.0
Total fatty acids, g	19.6	11.1	28.2	24.2	14.2	18.0	7.43	19.9	20.1	11.2
UFAs, g	15.9	8.58	19.5	14.2	11.4	15.1	6.66	15.5	11.9	9.87
SFAs, g	3.68	2.52	8.68	10.0	2.79	2.86	0.77	4.45	8.20	1.33
Cholesterol, mg	78.2	93.9	93.3	78.6	78.2	82.8	78.0	130	88.6	65.0
Sodium, mg	1007	1041	777	2212	545	984	562	788	2181	546

[1]The calorie and carbohydrate values in the stir fry entrées were lower, compared to the other entrées, because rice was not included in the recipe analyses, and rice was not provided to participants.

Abbreviation: UFAs, unsaturated fatty acids.

Supplemental Table 2. Total and incremental $AUC_{0\text{-}120\ min}$ for glucose, insulin, and glucoregulatory hormones at baseline and end of each condition in the evaluable analysis sample.[1]

Variables	Baseline[2]	Beef[3]	Poultry[3]	*P* Value[4]
$tAUC_{0\text{-}120\ min}$ glucose, [min x (mg/dL)]	15,060 (13,223, 17,153)	14,627 (14,060, 15,216)	14,899 (14,322, 15,499)	0.436
$iAUC_{0\text{-}120\ min}$ glucose, [min x (mg/dL)]	1175 (328, 4217)	627 (314, 1251)	837 (419, 1671)	0.540
$tAUC_{0\text{-}120\ min}$ insulin, [min x (μIU/mL)]	4836 (3038, 7697)	4802 (4328, 5328)	4626 (4169, 5133)	0.494
$iAUC_{0\text{-}120\ min}$ insulin, [min x (μIU/mL)]	3859 (2354, 6327)	3844 (3404, 4341)	3681 (3260, 4157)	0.482
$tAUC_{0\text{-}120\ min}$ C-peptide, [min x (pg/mL)] x 10^{-3}]	660 (503, 867)	651 (612, 692)	680 (639, 723)	0.173
$iAUC_{0\text{-}120\ min}$ C-peptide, [min x (pg/mL)] x 10^{-3}]	244 (72.0, 825)	306 (269, 347)	327 (288, 371)	0.396
tAUC $C\text{-}peptide_{0\text{-}120\ min}$/tAUC $glucose_{0\text{-}120\ min}$, (pg/mL)/(mg/dL)	43.8 (33.8, 56.8)	44.5 (41.8, 47.4)	45.6 (42.8, 48.6)	0.440
iAUC $C\text{-}peptide_{0\text{-}120\ min}$/iAUC $glucose_{0\text{-}120\ min}$, [(min x (pg/mL))/(min x (mg/dL))]	195 (36.4, 1042)	370 (240, 570)	305 (198, 470)	0.380
$tAUC_{(0\text{–}120\ min)}$ glucagon, [min x (pg/mL)] x 10^{-3}]	24.6 (10.6, 57.0)	22.2 (16.1, 30.5)	26.3 (19.1, 36.2)	0.194
$iAUC_{0\text{-}120\ min}$ glucagon, [min x (pg/mL)] x 10^{-3}]	8.40 (2.89, 24.5)	8.49 (6.07, 11.9)	10.4 (7.45, 14.6)	0.298
$tAUC_{0\text{-}120\ min}$ GLP-1, [min x (pg/mL)] x 10^{-3}]	52.4 (25.2, 109)	53.8 (44.8, 64.5)	61.3 (51.1, 73.5)	0.166

$iAUC_{0\text{-}120\ min}$ GLP-1, [min x (pg/mL)] x 10^{-3}]	11.2 (2.95, 42.5)	13.8 (10.0, 18.9)	15.3 (11.1, 21.0)	0.479
$tAUC_{0\text{-}120\ min}$ GIP, [min x (pg/mL)] x 10^{-3}]	440 (318, 608)	429 (394, 466)	451 (415, 490)	0.331
$iAUC_{0\text{-}120\ min}$ GIP, [min x (pg/mL)] x 10^{-3}]	252 (70.5, 900)	324 (275, 381)	320 (272, 377)	0.892

[1]Sample size: *n* = 24 for each condition.
[2]Results reported are GM (-1 SD, +1 SD).
[3]Results reported are LSGM (95% CI) for end-of-condition.
[4]*P* values are for Beef vs. Poultry using repeated measures analysis of covariance with baseline as a covariate.
Abbreviations: CI, confidence interval; GIP, glucose-dependent insulinotropic polypeptide; GLP-1, glucagon-like peptide-1; GM, geometric mean; iAUC, incremental area under the curve; LSGM, least squares geometric mean; tAUC, total area under the curve.
